# Diseases of the Kidney

**Published:** 1905-03-18

**Authors:** 


### DISEASES OF THE KIDNEY.

*(Concluded from page 425.)*

A Difficulty in Diagnosis.—The difficulty of distinguishing clinically some cases of diabetes insipidus from nephritis is shown by J. Burton Blaikie.[9] A girl of 11 was admitted suffering from thirst and polyuria, the urine amounted to 90 oz. daily of a specific gravity of about 1005, and contained a trace of albumen. She had always suffered from thirst and was at times voracious. After a fortnight gastric pain came on and the patient sank into a comatose condition and died. The kidneys were found to be in a state of chronic nephritis, extremely contracted and atrophied, and of a marked yellowish white colour.

Cystic Disease.—Boinet and Rayband[10] discuss the congenital cases. They think that long survival only occurs when the lesion is unilateral. Frequently there is malformation of the ureters and of various other organs, such as the liver. Virchow thought that these cystic kidneys are due to inflammatory occlusion of the channels in fœtal life. Laveran held the disease to be due to a new growth, and others that it is due to dilatation of the tubes from weakness of the walls. Strictures causing retention cysts, or the persistence of remnants of the Wolffian bodies have also been suggested as causes. The writers, like Luzzato, deny all these theories and think it due to a toxic hyperplasia of the interstitial tissue which affects the kidney chiefly in the medullary or last developed part of the organ. Victor Milward[11] referring to the symptoms of cystic kidney in the adult, mentions that the disease is never seen between the tenth and thirtieth year. In this period cysts are not to be found even post-mortem. Hence it has been said that the cysts are not congenital, taking on rapid growth in middle life, but really commence then. There is first a progressive enlargement, with no symptoms; next a period of months, or years, in which dull, aching, and sometimes paroxysmal pain is felt, with hæmaturia, albumen, leucocytes, and even pus in the urine which is of low specific gravity. There may be good general health till near the end of this stage, when uræmia, lessened urea, and brain complications occur. Heart symptoms are late, even though the kidneys are so large that they can be palpated through the abdominal walls. It may, however, be remarked upon the above paper, that it is not quite correct to state that cystic kidneys are never found in the second and third decades of life. Instances are not unknown. Two or three specimens from such cases at least exist in the museums of this country, but there are other reasons for thinking that adult cystic kidneys are not commonly developed from congenital ones.

[9] Lancet, Sept. 24. [10] Birm. Med. Rec., Nov. [11] Ibid, Aug.

